# Factors Associated with e-Health Literacy Among Older Adults in Klang Valley, Malaysia: A Cross-Sectional Survey

**DOI:** 10.3390/healthcare14183129

**Published:** 2026-09-21

**Authors:** Lubna Samer Allawi, Thet Htoo Pan, Myo Nyein Aung, Lwin Mie Aye

**Affiliations:** 1 Public Health and Community Medicine Department, School of Medicine, IMU University, Kuala Lumpur 57000, Malaysia; allawilubna@gmail.com; 2 Department of Global Health Research, Graduate School of Medicine, Juntendo University, Tokyo 113-8421, Japan; myo@juntendo.ac.jp; 3 Faculty of International Liberal Arts, Juntendo University, Tokyo 113-8421, Japan; 4 Advanced Research Institute for Health Sciences, Juntendo University, Tokyo 113-8421, Japan; 5 Jeffrey Cheah School of Medicine and Health Sciences, Monash University Malaysia, Subang Jaya 47500, Malaysia

**Keywords:** eHealth literacy, older adults, sociodemographic factors, digital health, health inequalities, Klang Valley, Malaysia

## Abstract

**Background**: In recent years, artificial intelligence (AI)-integrated platforms have emerged, providing access to health information and extending beyond information retrieval to include personalized health monitoring and decision support. However, many older adults still have limited digital health literacy, placing them at risk of increased health inequalities and missing opportunities for better health outcomes. Despite the growing importance of digital health information, evidence on eHealth literacy among older adults in Malaysia remains limited, particularly regarding the sociodemographic factors associated with differences in eHealth literacy. Understanding these associations is important for identifying population groups that may be more vulnerable to disparities in accessing, understanding, and evaluating online health information. Therefore, this study aimed to assess the association between eHealth literacy levels and sociodemographic factors among older adults in Klang Valley, Malaysia. **Methods**: A community survey recruited older adult participants in Klang Valley, Malaysia. An internationally validated instrument, the eHealth Literacy Scale (eHEALS), was used to measure the current eHealth literacy level of older adults. Chi-square tests and bivariate and multivariable logistic regression analyses were conducted to identify factors associated with eHealth literacy. **Results**: The mean eHEALS score was 27.29 ± 7.61 (range: 8–40), with the majority of participants demonstrating moderate or high perceived eHealth literacy. The bivariate analysis showed that age, ethnicity, education level, pre-retirement occupation, household income, language ability, and English proficiency were significantly associated with eHealth literacy. The multivariable analysis revealed that participants aged 70 years and above and those with a lower income had higher odds of low eHealth literacy (*p* < 0.05). **Conclusions**: This study demonstrates that eHealth literacy among older adults in Klang Valley is at a moderate level and is significantly associated with key sociodemographic factors. These findings underscore the need for targeted interventions and inclusive digital health strategies to improve eHealth literacy and reduce health inequalities among older adults in Malaysia.

## 1. Introduction

The aging population is increasing globally and in Malaysia, creating growing health information needs among older adults. Older adults often face multiple health conditions and need to make frequent health-related decisions. Therefore, access to reliable, understandable, and actionable health information is increasingly important for healthy aging and self-management. Malaysia is currently undergoing a demographic shift toward an aging population, with predictions that almost 15% of the country’s population will be aged 60 years and above by 2030 and that 15% of the population will be aged over 65 years by 2050 [1]. Older adults in Malaysia face challenges in accessing and obtaining digital health information, often as a result of their limited familiarity with digital tools, low educational levels, and physical and cognitive limitations [2,3].

The availability of digital health-related information has enabled users to obtain reliable knowledge regarding their symptoms and health conditions, which allows them to explore prevention strategies and treatment options with just a few clicks [4]. Digital health technologies have improved health management by making healthcare services more personalized and efficient [5]. Innovations such as wearable fitness trackers, telehealth, and mobile health applications have enhanced individuals’ ability to make informed health choices and manage their health conditions effectively [6]. The significance of these health tools has increased, particularly during the COVID-19 pandemic, which emphasizes the importance of such innovations in managing public health crises [7]. Following the COVID-19 pandemic, populations relied on online sources and digital devices to carry out daily tasks. These tools have facilitated a global shift toward digital health services by ensuring healthcare accessibility while minimizing physical interaction [8]. Nevertheless, the effective use of these tools requires a certain level of eHealth literacy.

eHealth literacy among older adults may be influenced by a range of sociodemographic and social factors. Age is an important consideration, as increasing age may be associated with reduced familiarity with digital technologies and greater difficulty adapting to rapidly changing digital environments. Education attainment may also influence eHealth literacy by shaping an individual’s ability to understand, interpret, evaluate, and apply health information obtained from electronic sources. Socioeconomic circumstances may further contribute to differences in eHealth literacy, as individuals with higher household incomes may have greater opportunities to access digital devices, reliable internet services, and other resources that facilitate engagement with digital health information. Previous occupation may similarly reflect differences in exposure to technology, information resources, and digital environments across the life course [9]. Social and demographic characteristics may also shape opportunities to acquire and use digital health skills. Living arrangements may influence access to informal assistance, particularly when older adults rely on family members or other household members for support when using digital devices and accessing online information. Ethnicity may also be relevant in the Malaysian context because ethnic groups may differ in language, cultural practices, socioeconomic circumstances, and access to health information that is linguistically and culturally appropriate [10]. These factors suggest that eHealth literacy should be considered within the broader context of social and sociodemographic inequalities rather than solely as an individual-level digital skill.

One of the main barriers to fully benefiting from new health technologies is inadequate electronic health (eHealth) literacy. The potential of digital health tools to transform healthcare cannot be fully realized without adequate knowledge and skills to use them effectively [11]. As defined by Norman and Skinner, eHealth literacy is “the ability to seek, find, and understand health information from electronic sources and apply the information gained to address or resolve health issues” [12]. It is a newly developed concept of digital literacy that has arisen as an important factor influencing health-related behaviors, at a time when digital technology is being incorporated into healthcare services and online health information is widely available [13].

Moreover, the high prevalence of English-language health content on health websites and digital platforms limits understanding and usability for non-English speakers. Access to English-language health information does not necessarily ensure that individuals can understand and use such information effectively [14]. This issue may be particularly relevant among older adults, as limited English proficiency can reduce their ability to evaluate, interpret, and apply online health information. Previous studies have reported that individuals who primarily use languages other than English may experience greater difficulty in understanding online health information, lower confidence in evaluating its quality, and greater frustration during online health information seeking [15]. In Malaysia, the limited availability of localized and linguistically appropriate digital health resources may further restrict meaningful access to online health information, particularly among older adults who primarily communicate in Malay or other languages.

In recent years, artificial intelligence (AI) platforms have also emerged as important tools for accessing health information, extending beyond information retrieval to include personalized health monitoring and decision support. AI-powered chatbots, virtual assistants, and remote monitoring systems are increasingly being used to support health education, symptom assessment, disease management, and preventive care, providing rapid responses to health-related queries and potentially improving access to health information for diverse populations [16]. Wearable technologies can continuously collect health-related data, such as physical activity, heart rate, and sleep patterns, while AI algorithms can analyze these data to provide personalized feedback and identify potential health risks [17]. AI-based systems may also support older adults through functions such as health monitoring, safety supervision, and emotional and cognitive assistance [18]. However, the skills required to access and use general online health information are not necessarily equivalent to AI-specific literacy, which may additionally involve understanding the capabilities and limitations of AI systems and critically evaluating AI-generated information.

Older adults often face challenges that limit their online access and confidence in effectively utilizing digital health technologies. These challenges include cognitive decline, visual impairment, and limited experience with technology. Despite the wide range of health-related information available online, many older adults still have limited digital literacy, placing them at risk of greater health inequalities and missing opportunities for better health outcomes. Previous studies in Malaysia have reported that older adults may experience difficulties in evaluating the quality and relevance of health information obtained online [19]. In Malaysia, previous studies have found that, while older adults may be able to seek and find health information online, many report limited confidence in evaluating the quality of the health information obtained and in applying it to their personal needs [13]. This issue becomes more concerning given the high demand for and reliance on digital tools and platforms for health management.

As the Malaysian government continues to invest in digital health platforms, the potential exclusion of older adults from digital health initiatives remains a critical concern. However, despite these challenges, there remains limited evidence on the factors associated with eHealth literacy among older adults in Malaysia, particularly within the Klang Valley context. Existing research has largely focused on general access to digital health information and barriers to digital technology use, while less is known about how sociodemographic characteristics are associated with older adults’ perceived ability to seek, evaluate, and use online health information. Addressing this gap is important for informing inclusive digital health strategies that can better support older adults in Malaysia.

Understanding current eHealth literacy levels and their sociodemographic correlates among older adults is essential to ensure equitable access to digital health resources and to support healthy aging within Malaysia’s increasingly digitalized healthcare system. The findings of the present study will provide researchers and public health professionals with baseline evidence on the level of perceived eHealth literacy and sociodemographic correlates among a sample of Malaysian older adults. Establishing this baseline is important, as digital health technologies continue to evolve, including increasing integration of artificial intelligence (AI) into health information and healthcare services. While the present study focuses on general perceived eHealth literacy, future research can build on these findings by examining AI-specific competences, including older adults’ ability to critically evaluate, interpret, and appropriately use AI-generated health information and AI-enabled health technologies [20]. Klang Valley was selected as the study setting due to its high population density, advanced digital infrastructure, and wide availability of digital health services, making it an appropriate context for examining sociodemographic factors associated with eHealth literacy among the older population.

Therefore, this study aimed to assess eHealth literacy among older adults in Klang Valley, Malaysia, and to examine its associations with sociodemographic characteristics. The objectives of the study were to (1) assess the level of eHealth literacy among older adults residing in Klang Valley, Malaysia, and (2) examine the association between sociodemographic factors and eHealth literacy.

## 2. Methods

### 2.1. Study Settings

This was a quantitative, cross-sectional study conducted to examine the factors associated with eHealth literacy among community-dwelling older adults in Klang Valley, Malaysia. The study involved older adults aged 60 years and above, and it employed a structured questionnaire to collect participants’ sociodemographic and health-related information. The research study was conducted in Klang Valley, Malaysia, which is an urban agglomeration centered in the federal territories of Kuala Lumpur and Putrajaya. The researcher visited specific locations that have high concentrations of older adults. Participants were recruited using purposive and convenience sampling from selected community settings in Klang Valley, such as senior clubs, health clinics, community centers, and places of worship where older adults frequently gather.

### 2.2. Sample Size and Sampling Method

There are an estimated 614,527 eligible individuals residing in Klang Valley, Malaysia, which accounts for around 28.0% of the country’s total older adult population [21]. This population represents a mix of urban settings and socioeconomic backgrounds, as Klang Valley is Malaysia’s most populous urban region. It encompasses Kuala Lumpur, Putrajaya, and surrounding areas in Selangor and serves as a major economic, administrative, and technological hub. Its combination of highly developed cities and semi-urban areas, along with diverse socioeconomic characteristics, makes it a suitable location for this study [22].

The minimum sample size was calculated a priori using the standard formula for estimating a single population proportion, n = Z^2^p(1 − p)/e^2^, where Z = 1.96 corresponds to a 95% confidence level; *p* = 0.5 is assumed because the prevalence of low eHealth literacy among older adults in Klang Valley was unknown, and this value yields the most conservative estimate; and e = 0.05 represents a 5% margin of error. This yielded a minimum of 384 participants. A 20% allowance for non-response was added, giving a target sample of 480 participants. The calculation was based on the first study objective, namely, estimating the level of eHealth literacy in this population with a specified precision; it was not a power calculation for the multivariable logistic regression, and no assumed effect size, statistical power, or number of predictors was specified for that analysis.

This was the initial baseline study employing a non-probability sampling approach. Eligible participants were recruited during the defined data collection period based on predetermined inclusion and exclusion criteria. A total of 200 participants with complete baseline data were included in the analysis. The achieved sample was used to provide an initial assessment of the characteristics of the study population and factors associated with the outcome of interest. Given the exploratory nature of the baseline study and the non-probability sampling design, the findings should be interpreted within the context of the study population and should not be generalized to the wider population without further validation.

Recruitment took place over the predefined data collection period from September to December 2025. Within this period, 200 eligible older adults provided written informed consent and were enrolled. All 200 completed the questionnaire in full: there were no withdrawals and no missing data, so all 200 were included in the analysis.

This study was carried out from September 2025 to December 2025. Eligible participants were Malaysian adults aged 60 years and above, including both males and females, who were able to access or use digital devices. Individuals with severe physical or cognitive impairments that could limit participation, as well as institutionalized older adults, were excluded from the study. Older adults were approached in different public settings within Klang Valley, such as community centers, health clinics, worship places, senior clubs, and other areas where older people commonly gather. This method was chosen as it enabled efficient and practical recruitment of participants who met the study’s inclusion criteria within the study period.

### 2.3. Research Instruments

In this study, data were collected using a validated self-administered questionnaire, which comprised 22 items across two sections, designed to capture information relevant to the study variables. The questionnaire was administered in both English and Malay, and each participant chose the language in which they completed it. The eHealth literacy items were taken from the original 8-item eHEALS of Norman and Skinner [12]. The full questionnaire, including the eHEALS items, was translated into Malay for the present study. A forward translation into Malay was carried out by a bilingual translator, followed by an independent back-translation into English. The original and back-translated versions were compared to identify discrepancies in wording and meaning, and the final Malay version was reviewed for clarity and cultural appropriateness for the Malaysian older adult population.

A Malay version of the eHEALS has previously been translated and validated in a Malaysian primary care population [23]. That version was not used in the present study; the Malay questionnaire described above was translated independently from the original English instrument. The implications of this are considered in Section 4.7.

Face validity was assessed through a pilot evaluation involving 20 participants. Participants were asked to provide feedback on the clarity and understanding of the items and to identify any items that were confusing or difficult to understand. The feedback indicated that the questionnaire items were clear, understandable, and relevant, and no modifications to the items were required. The final questionnaire was subsequently used for the main data collection. The internal consistency of the eHEALS was assessed using Cronbach’s alpha. The eight-item eHEALS demonstrated excellent internal consistency in the present study (n = 200, Cronbach’s α = 0.951), with corrected item–total correlations ranging from 0.718 to 0.876. This is comparable to the value of 0.970 reported for a previously validated Malay version of the scale [23], supporting the internal consistency of the translation used here.

#### 2.3.1. Section A: Sociodemographic Characteristics

Sociodemographic information of the participants was collected to assess their background and general sociodemographic status. Name, age (≥60 years), gender (male or female), ethnicity (Malay, Chinese, or Indian), and living arrangements were among the questions asked. Additional questions were included to obtain further details about the participants’ current monthly household income (RM < RM2500–≥RM10,960), current health status, academic qualifications, occupation status, and previous occupation prior to retirement. Moreover, language ability and English proficiency were included as separate candidate predictors because they were intended to capture different aspects of linguistic access to online health information. Language ability assessed whether participants were able to use English, whereas English proficiency assessed the degree of English ability, including the capacity to understand more complex vocabulary and health-related terminology. Both variables were considered relevant because the ability to access English-language health information does not necessarily imply sufficient proficiency to understand health and clinical terminology. These sociodemographic factors were obtained in order to understand the participants’ personal and socioeconomic backgrounds, which may be associated with their eHealth literacy levels.

#### 2.3.2. Electronic Health Literacy (eHEALS)

This section covers the 8-item eHealth Literacy Scale (eHEALS) by Norman and Skinner (2006) [12], which examines an individual’s perceived ability to find, assess, and utilize online health information. Responses are graded on a 5-point Likert scale (1 = strongly disagree to 5 = strongly agree), generating scores from 8 to 40, with higher scores indicating higher eHealth literacy. High scores indicate a high level of eHealth literacy, while low scores indicate low eHealth literacy and confidence in using online health information. The scores of this scale were categorized as follows:

- Low eHealth literacy: 8–20;
- Moderate eHealth literacy: 21–30;
- High eHealth literacy: 31–40.

The total eHEALS score ranged from 8 to 40 and was initially categorized into low (8–20), moderate (21–30), and high (31–40) levels for descriptive analysis. For the multivariable analysis, eHealth literacy was dichotomized into low eHealth literacy (8–20) and moderate-to-high eHealth literacy (21–40). This approach was used to distinguish participants reporting low perceived eHealth literacy from those reporting at least moderate perceived eHealth literacy, allowing the analysis to focus on the factors associated with a potentially more vulnerable group. The 8–20 band used here to define low eHealth literacy corresponds to the “inadequate” eHealth literacy category identified in the psychometric threshold analysis of the eHEALS by Wångdahl et al. [24], which supports the 20/21 cut-off applied to the dichotomized outcome. The moderate (21–30) and high (31–40) bands were used for descriptive purposes only and do not correspond to the upper thresholds proposed in that study; because the multivariable analysis contrasted low with moderate-to-high eHealth literacy, only the 20/21 threshold was used analytically. Binary logistic regression was therefore used to estimate the odds of low perceived eHealth literacy compared with moderate-to-high perceived eHealth literacy. Dichotomizing the three-level eHEALS measure necessarily reduces the information contained in the original continuous score and may obscure differences between participants with moderate and high perceived eHealth literacy; this was therefore considered when interpreting the findings. Participants with scores of 8–20 were categorized as having low eHealth literacy, while those with scores of 21–40 were categorized as having moderate-to-high eHealth literacy.

The eHEALS has demonstrated strong psychometric properties, with high internal consistency reported in prior studies (Cronbach’s alpha ranging from 0.88 to 0.93) and appropriate construct validity across many populations, including older adults. The scale efficiently assesses self-perceived eHealth literacy and confidence in engaging with electronic health information; thus, it is an ideal assessment tool for older adults due to its clarity and relevance.

The 8-item eHealth Literacy Scale (eHEALS) by Norman and Skinner (2006) [12] was used to assess participants’ perceived ability to seek, find, assess, and apply health information obtained from electronic sources. The scale therefore primarily captures self-reported or perceived eHealth literacy and confidence in engaging with online health information rather than objectively measured digital competence. It does not specifically assess AI literacy, including the ability to evaluate AI-generated health information, understand algorithmic processes, or assess trustworthiness and potential biases in AI outputs. Accordingly, the present study focuses on general perceived eHealth literacy, while implications regarding AI-enabled health technologies are considered in a broader and future-oriented context rather than as outcomes directly measured by the eHEALS.

### 2.4. Data Collection Procedure

Data were collected using a structured, self-administered questionnaire that was distributed to older adults residing in the Klang Valley region. Participants were recruited face to face at community centers, health clinics, senior clubs, and worship places using the purposive and convenience sampling method. Prior to participation, informed consent was obtained from all respondents interested in participating in the study. The questionnaire included two main sections, and most participants were able to complete it within approximately 8–10 min. Assistance from the researcher was provided to those with reading difficulties to ensure accurate responses, and all responses were kept confidential and securely stored for data protection purposes.

### 2.5. Data Analysis

In this study, Statistical Package for the Social Sciences (SPSS) version 30.0 was used to analyze the data obtained.

#### 2.5.1. Descriptive Statistics

Descriptive statistics were employed to summarize the participants’ sociodemographic characteristics and eHealth Literacy Scale (eHEALS) scores. Categorical variables are reported as frequencies and percentages, whereas continuous variables are reported as means with standard deviations.

#### 2.5.2. Inferential Statistics

Inferential statistical analyses were conducted to examine the relationships between eHealth literacy and independent variables. Chi-square tests and bivariate analyses were performed to assess associations between eHealth literacy and sociodemographic variables. Eight variables with a *p*-value of <0.25 in the bivariate analyses—age, ethnicity, education level, previous job, monthly income, living arrangement, language ability, and English proficiency—were considered candidate variables for inclusion in the binary logistic regression model to determine the independent predictors of eHealth literacy. The *p* < 0.25 threshold was used as a screening criterion to avoid prematurely excluding potentially relevant variables from the multivariable analysis. The eight candidate variables were subsequently entered into a binary logistic regression model using the Forward Likelihood Ratio (Forward LR) method, with variables retained in the final model based on their contribution to the model. Since the dependent variable (eHealth literacy) was dichotomized into low and moderate/high categories, binary logistic regression was considered appropriate. A *p*-value of less than 0.05 was considered indicative of statistical significance in the final model.

To assess whether the findings were dependent on the categorization of the outcome, a sensitivity analysis was performed in which the total eHEALS score was analyzed as a continuous variable (range 8–40) using linear regression. Crude associations were estimated for each sociodemographic variable separately, and all sociodemographic variables were then entered simultaneously into a multivariable linear regression model. Multicollinearity was assessed using tolerance and variance inflation factor (VIF) values. The results of this sensitivity analysis are presented in Supplementary Table S1.

### 2.6. Ethical Considerations

This research was approved by the IMU Joint-Committee on Research and Ethics (Approval Code 4.16/JCM-316/2025; Approval Date: 22 July 2025). Privacy and confidentiality were the first ethical concerns to be addressed prior to the beginning of the study. Accordingly, written consent forms and information pages were given to each participant. Confidentiality extended to data access; only student researchers and supervisors had access to all collected data, including raw SPSS files and questionnaire information.

## 3. Results

### 3.1. Sociodemographic Characteristics of the Respondents

In this study, a total of 200 participants were successfully recruited and included in the final analysis. Table 1 reveals that the majority of participants were aged 60–69 years (72%), female (60%), and of Malay ethnicity (52.5%). Most participants had attained at least a secondary level of education, with 28.5% holding a university degree, 27.5% having completed secondary school, and 21.5% possessing a diploma, while only a small proportion reported postgraduate qualifications. More than half of the participants were retired (56%), followed by those who were employed full time (15.5%), housewives (11.5%), self-employed (8%), employed part time (3.5%), and unemployed (5%). Among retired participants, previous employment consisted predominantly of professional or managerial roles (33%), followed by clerical or administrative roles (13%), with smaller proportions reporting technical/skilled, unskilled, sales or service, self-employment, or other occupations. Most participants reported a monthly household income between RM2500 and RM4849 (30%) or RM4850 and RM10,959 (31%), and 36.5% received a pension. Regarding living arrangements, 50.5% lived with children, relatives, or other family members, while 32.5% lived with their spouse only.

Health status assessment revealed a high burden of cardiometabolic conditions. Hypertension was the most commonly reported condition (43%), followed by high cholesterol (36.5%) and diabetes (26.5%). Heart disease was reported by 10.5% of participants, while cancer (2.5%) and chronic respiratory diseases (1.5%) were less prevalent. Notably, 31% reported having no diagnosed health conditions. As multiple responses were permitted, a total of 303 responses were recorded for this item. In terms of language use, Malay was the most commonly spoken language (94%), followed by English (82.5%), indicating a high level of bilingualism. Other languages reported included Mandarin (24.5%), other Chinese dialects (20%), and Tamil (14%). Despite widespread English use, proficiency levels varied: 38% reported advanced proficiency, 30.5% intermediate, 15.5% basic, and 8% no proficiency, suggesting that limited English proficiency may still pose a barrier to accessing English-dominant online health information.

### 3.2. eHealth Literacy Among Participants

The eHealth Literacy Scale (eHEALS) was used to assess participants’ perceived ability to seek, access, evaluate, understand, and use health information obtained from electronic sources. The eHEALS scores ranged from 8 to 40, with a mean score of 27.29 ± 7.61 (Table 2). The distribution of eHealth literacy levels among participants is presented in Table 3. Of the 200 participants, 42.5% (n = 85) had high eHealth literacy, 41.0% (n = 82) had moderate eHealth literacy, and 16.5% (n = 33) had low eHealth literacy. Overall, 83.5% of participants had moderate or high perceived eHealth literacy.

### 3.3. Association Between Sociodemographic Factors and e-Health Literacy (Bivariate Analysis)

As shown in Table 4, the bivariate analysis found that several sociodemographic factors were significantly associated with the levels of eHealth literacy among the study participants. Higher eHealth literacy was observed among participants who were aged between 60 and 69 years; who were female; who were Malay or Chinese; who had higher education qualifications; and whose main occupation before retiring involved professional, technical, or clerical roles. Moreover, participants with a higher monthly household income and English-language ability also showed higher levels of eHealth literacy. Significant associations were observed between eHealth literacy and age (*p* < 0.002), ethnicity (*p* < 0.04), highest academic qualifications (*p* < 0.001), main job before retiring (*p* < 0.001), household income (*p* < 0.001), language ability (*p* < 0.001), and English proficiency (*p* < 0.001). In contrast, gender, occupation status, receiving a pension, living arrangements, and the presence of chronic disease were not significantly associated with eHealth literacy (*p* > 0.05). These findings also contribute to addressing the study’s second specific objective, which was to examine the association between sociodemographic factors and eHealth literacy.

### 3.4. Sociodemographic Factors Associated with e-Health Literacy (Logistic Regression Analysis)

Table 5 presents the multivariable logistic regression analysis of factors associated with eHealth literacy. Eight candidate predictors identified in the bivariate analyses using the *p* < 0.25 screening criterion—age, ethnicity, education level, previous job, monthly income, living arrangements, language ability, and English proficiency—were entered into the multivariable model using the Forward Likelihood Ratio (Forward LR) method. The Forward LR procedure subsequently retained variables that contributed significantly to the final model. Statistical significance was defined as *p* < 0.05. Prior to modeling, multicollinearity was assessed using variance inflation factor (VIF) and tolerance values, and no issues were detected. Interaction terms between key predictors were tested and were not significant. The model fit was evaluated using the Hosmer–Lemeshow goodness-of-fit test, which indicated an adequate fit (χ^2^ = 6.67, df = 8, *p* = 0.57). The explained variance of the model was assessed using Cox and Snell R^2^ = 0.39 and Nagelkerke R^2^ = 0.67, indicating that the predictors together explained a substantial proportion of the variability in eHealth literacy.

Several variables remained associated with eHealth literacy after the adjustment of cofounders. Participants aged ≥70 years had higher odds of low eHealth literacy than the reference age group, which was 60–69 years (adjusted OR = 6.02; 95% CI = 1.14–31.57; *p* = 0.03). Education level was also significantly associated with eHealth literacy. Participants with tertiary education had higher adjusted odds of low eHealth literacy than those with ≤secondary education (adjusted OR = 9.28; 95% CI = 1.12–76.35; *p* = 0.03). This direction is opposite to that observed in the unadjusted analysis, and the very wide confidence interval indicates substantial imprecision; this estimate should therefore be interpreted with caution (see Section 3.5 and Section 4.3). Among the income categories, participants with an average monthly income of ≥RM10,960 were less likely to have low eHealth literacy than those earning <RM2500 (adjusted OR = 0.02; 95% CI = 0.001–0.53; *p* = 0.01). Other factors, including gender, ethnicity, and occupation, were not significant after adjustment. However, some estimates had wide confidence intervals, reflecting limited precision, possibly due to small sample sizes within certain subgroups and variability in the data; therefore, these results should be interpreted with caution.

### 3.5. Sensitivity Analysis Using the Continuous eHEALS Score

To examine whether the associations observed in the logistic regression were dependent on the dichotomization of the outcome, the total eHEALS score was reanalyzed as a continuous variable using linear regression (Supplementary Table S1). In the crude analyses, higher eHEALS scores were associated with tertiary education (B = 6.27; 95% CI: 4.21 to 8.33; *p* < 0.001), a higher household income (B = 2.76; 95% CI: 1.78 to 3.73; *p* < 0.001), English-language ability (B = 7.49; 95% CI: 4.90 to 10.09; *p* < 0.001), and higher English proficiency (B = 4.10; 95% CI: 3.10 to 5.10; *p* < 0.001), whereas age ≥ 70 years was associated with lower scores (B = −4.60; 95% CI: −6.88 to −2.32; *p* < 0.001).

The multivariable linear model, in which all sociodemographic variables were entered simultaneously, explained 35.9% of the variance in the eHEALS scores (R^2^ = 0.359; adjusted R^2^ = 0.321; F(11, 188) = 9.55; *p* < 0.001), and no multicollinearity was detected (all VIF ≤ 2.42). Age ≥ 70 years (B = −2.77; 95% CI: −4.97 to −0.57; *p* = 0.014), ethnicity (B = −1.62; 95% CI: −2.88 to −0.37; *p* = 0.012), and higher English proficiency (B = 2.25; 95% CI: 0.77 to 3.72; *p* = 0.003) remained independently associated with the eHEALS score, whereas the association with higher household income was attenuated to borderline significance (B = 1.03; 95% CI: −0.04 to 2.10; *p* = 0.058).

The association with age was consistent in both direction and statistical significance across the dichotomized and continuous analyses, and the association with household income was in the same direction in both, although with only borderline significance in the continuous analysis (*p* = 0.058). This supports the broad robustness of these findings to the categorization of the outcome. For education, the two analyses diverged: tertiary education was associated with a higher eHEALS score in the crude analysis (B = 6.27; *p* < 0.001), and the direction of this association remained positive, although attenuated and no longer statistically significant, after adjustment (B = 1.28; 95% CI: −1.14 to 3.70; *p* = 0.297). The continuous analysis therefore did not reproduce the increased adjusted odds of low eHealth literacy associated with tertiary education observed in the logistic model, which suggests that the latter estimate is unstable; this is considered further in Section 4.3.

## 4. Discussion

The findings of this study indicate that several sociodemographic factors were associated with eHealth literacy among older adults in Klang Valley, Malaysia. In particular, age, education level, and average monthly household income were significantly associated with eHealth literacy after adjustment for other variables in the multivariable analysis, and they were identified as key factors associated with participants’ perceived ability to access, understand, and use online health information. These findings are particularly relevant in the context of the increasing digitalization of healthcare, as older adults are increasingly required to access and engage with health information through digital platforms. As AI-driven health platforms, telehealth services, and digital decision support tools become more common, general eHealth literacy may have broader relevance to engagement with increasingly digitalized healthcare environments. However, the present study assessed general perceived eHealth literacy using the eHEALS and did not specifically measure AI literacy or the ability to evaluate AI-generated health information. Therefore, the findings should not be interpreted as direct evidence of older adults’ readiness to use or evaluate AI-enabled health technologies. Rather, they provide evidence of sociodemographic differences in perceived general eHealth literacy as a baseline for future research examining how these differences may relate to engagement with emerging AI-mediated digital health environments.

### 4.1. e-Health Literacy Among Older Adults in Klang Valley, Malaysia

According to a meta-analysis, the pooled mean eHEALS score among older adults was 21.45, which is below the reported adequacy threshold of ≥32 [19]. In this study, the mean eHealth literacy score was 27.29 ± 7.61. Although this mean remains below the reported adequacy threshold, it is higher than that reported in previous studies. These findings suggest that, despite variability in eHealth literacy levels, the overall mean eHealth literacy score among participants fell within the moderate range.

### 4.2. Age

This study found that increasing age was statistically associated with low levels of eHealth literacy. Participants aged ≥70 years had significantly higher odds of low eHealth literacy than the reference age group of 60–69 years (*p* = 0.03); however, the wide confidence interval indicates substantial uncertainty around the magnitude of this association. The findings are consistent with existing evidence indicating that increased age is associated with lower digital competence and lower confidence in understanding and using online health information [25]. The lower perceived eHealth literacy observed among older participants may reflect greater challenges in navigating online platforms and feeling confident when assessing health information. Prior research has repeatedly shown that older adults may experience greater difficulty adopting and using digital health technologies, which may restrict their engagement with online health resources [26]. However, these findings should be interpreted in relation to perceived eHealth literacy, as the present study did not objectively assess participants’ digital skills or their demonstrated ability to evaluate the quality or reliability of online health information. Nevertheless, lower perceived eHealth literacy among the oldest age group may contribute to greater vulnerability to digital exclusion as healthcare systems become increasingly digitalized.

### 4.3. Education Level

Education level was associated with eHealth literacy, but crude and adjusted analyses pointed in opposite directions. In the unadjusted analysis, tertiary education was associated with substantially lower odds of low eHealth literacy (COR = 0.31; 95% CI: 0.14–0.67; *p* = 0.003) and with an eHEALS score 6.27 points higher than secondary education or below (95% CI: 4.21 to 8.33; *p* < 0.001). These crude findings align with existing evidence suggesting that higher education improves an individual’s ability to assess and use online health information [27].

In the multivariable logistic model, however, tertiary education was unexpectedly associated with higher adjusted odds of low eHealth literacy (AOR = 9.28; 95% CI: 1.12–76.35; *p* = 0.03). The confidence interval spans almost two orders of magnitude, indicating that the magnitude of this estimate is very poorly determined. Two observations suggest that this reversal reflects instability in the model rather than a substantive finding. First, only 33 participants were classified as having low eHealth literacy, giving approximately four outcome events per candidate predictor, well below the conventional minimum of ten events per variable, so the model is vulnerable to sparse data bias. Second, the sensitivity analysis using the continuous eHEALS score did not reproduce the reversal: after adjustment for the same set of sociodemographic variables, the association between tertiary education and the eHEALS score remained positive, although attenuated and non-significant (B = 1.28; 95% CI: −1.14 to 3.70; *p* = 0.297; Supplementary Table S1). Taken together, these results indicate that the adjusted odds ratio for education should not be interpreted as evidence that higher education is associated with poorer eHealth literacy and that the association between education and eHealth literacy in this population requires confirmation in studies with larger samples and a greater number of outcome events.

In Malaysia, older people often face challenges in accessing and obtaining digital health information due to their limited exposure to digital tools, low educational levels, and physical and cognitive limitations [2,3]. Similar findings have been reported in studies conducted in China, Korea, and Thailand, where education, confidence level, income, and digital skills were identified as the main factors associated with low levels of eHealth literacy among older adults [10,28,29]. Furthermore, a Malaysian study conducted among primary healthcare patients demonstrated that higher education levels were significantly associated with improved eHealth literacy [23].

### 4.4. Average Monthly Household Income

Average monthly household income was also found to be a significant sociodemographic factor associated with eHealth literacy. Among income categories, participants with an average monthly income of ≥RM10,960 were less likely to have low eHealth literacy than those earning <RM2500 (*p* = 0.01). These findings are consistent with those of previous meta-analyses and systematic reviews that examined factors associated with eHealth literacy globally, which identified income as one of the key associated factors [26]. A higher income can facilitate greater access to digital devices, internet connectivity, and other resources required to engage with online health information. Conversely, individuals in lower-income groups may face financial constraints that limit access to digital devices and internet services, potentially contributing to digital health inequalities.

### 4.5. Language Proficiency and Digital Inclusion

Language proficiency may represent an additional dimension of digital inclusion in multilingual settings such as Malaysia, where access to understandable digital health information may depend partly on the language in which such information is available. Although most participants in the present study reported speaking English, levels of proficiency varied considerably, with 8% reporting no proficiency and 15.5% reporting only basic proficiency. This is relevant because a substantial proportion of online health information and digital health resources may be presented in English, potentially creating additional barriers for individuals with limited proficiency. In the bivariate analysis, English proficiency was significantly associated with eHealth literacy, with higher proficiency generally corresponding to a greater proportion of participants in the high eHealth literacy category. However, this association did not remain statistically significant for the individual proficiency categories in the multivariable model. Therefore, the findings should not be interpreted as evidence of a causal effect of English proficiency on eHealth literacy. Rather, they suggest that language may warrant further consideration as a dimension of digital inclusion, particularly in multilingual populations where access to understandable and culturally appropriate health information may vary according to language resources.

### 4.6. eHealth Literacy and the Digital Divide

The sociodemographic patterns observed in this study are best understood within the broader framework of the digital divide rather than as a set of individual deficits. The digital divide is commonly described as operating at successive levels: inequalities in material access to devices and connectivity, inequalities in the skills required to use them, inequalities in the range and autonomy of use, and ultimately inequalities in the benefits that people derive from digital technologies [30]. The associations observed here map onto this framework. Household income plausibly reflects material access; education and English proficiency reflect the skills and literacy resources required for meaningful use; and age reflects both generational differences in exposure to digital technology and the accumulated effect of these constraints over the life course. Comparable patterns have been reported among older adults internationally, where age, education, and income have consistently emerged as correlates of digital health literacy [28], and among older Americans, where limited health literacy and limited internet access have been shown to be closely intertwined sources of disadvantage [31].

Framing the findings in this way also directs attention beyond individual characteristics to the contexts in which older adults live. Area-level differences in socioeconomic composition and in the supply of health services contribute to systematic geographic variation in the use of health resources [32], and living environmental conditions have been shown to be associated with health outcomes among middle-aged and older adults in large population-based cohorts [33]. Considered alongside the present findings, this suggests that improving eHealth literacy among older adults in Klang Valley is unlikely to be achieved through individually targeted digital skills training alone and will also require attention to the affordability of devices and connectivity, the availability of health information in Malay and other local languages, and the support available to older adults from family and the community when they use digital health services.

### 4.7. Limitations

The eligibility criterion requiring participants to be able to access or use digital devices may have introduced selection bias. Older adults who are unable to access or use digital devices may represent a group at greater risk of digital exclusion and potentially lower eHealth literacy, but they were not represented in the study sample. Consequently, the findings may overestimate perceived eHealth literacy among the broader older population. In addition, recruitment through community centers, clinics, senior clubs, and places of worship may have preferentially included older adults who are more socially engaged and able to attend community settings. Therefore, the findings should not be generalized to socially isolated, homebound, institutionalized, or otherwise digitally excluded older adults in Klang Valley. In addition, the number of older adults who were approached but declined to give consent was not recorded, so a participation rate cannot be reported, and the characteristics of those who declined are unknown. If older adults who declined were less digitally engaged than those who took part, the perceived eHealth literacy reported here would be overestimated, compounding the selection effect of the digital device eligibility criterion.

The eHEALS was translated into Malay for this study rather than adopting the Malay version that had previously been translated and validated in a Malaysian primary care population [23]. The translated instrument showed high internal consistency in the present sample (Cronbach’s α = 0.951), comparable to the 0.970 reported for the previously validated version, but no formal psychometric validation of the present translation was undertaken, and direct comparability with studies using the established version is therefore limited. Future studies in Malaysia should consider adopting the previously validated Malay version.

An important limitation concerns the nature of the eHealth literacy measure used in this study. The eHEALS assesses individuals’ perceived confidence and self-reported ability to access, assess, and use electronic health information rather than objectively measured digital competence or demonstrated ability to evaluate the accuracy or quality of online health information. Furthermore, the eHEALS was developed to assess general eHealth literacy and does not specifically measure AI literacy, trust in AI systems, or the ability to critically evaluate AI-generated health information. Therefore, the findings should be interpreted as reflecting perceived general eHealth literacy rather than objective digital skills or readiness to use AI-enabled health technologies. Future research should incorporate objective measures of digital competence and AI-specific literacy to provide a more comprehensive assessment of older adults’ capabilities in contemporary digital health environments.

In addition, the categorization of eHEALS scores and subsequent dichotomization of the outcome into low versus moderate/high eHealth literacy may have resulted in some loss of information compared with an analysis of eHEALS scores as a continuous measure. Dichotomization may reduce variability and statistical power and may obscure differences between participants within the moderate and high eHealth literacy categories. This categorization, together with the relatively small number of participants classified as having low eHealth literacy, contributed to the sparse outcome events observed in the multivariable analysis. Future studies may consider analyzing eHEALS scores as a continuous measure or using alternative categorization approaches, where methodologically appropriate. To assess the impact of this categorization, a sensitivity analysis was conducted in which the eHEALS score was analyzed as a continuous variable (Supplementary Table S1). The associations for age and English proficiency were consistent across the two approaches, and the association for household income was in the same direction, although only of borderline significance in the continuous analysis, supporting the broad robustness of these findings; the association for education was not consistent, and this divergence is discussed in Section 4.3.

The achieved sample size was 200 participants, compared with the planned target of 480. The smaller sample may have reduced the precision of the estimates and limited the ability to detect associations, particularly among predictor categories with relatively few observations. Only 33 participants were classified as having low eHealth literacy, resulting in a relatively small number of outcome events for multivariable modeling. In addition, some predictor categories contained few observations, resulting in wide confidence intervals and imprecise estimates for some associations. Therefore, the multivariable findings should be interpreted cautiously, particularly the estimates with wide confidence intervals, and should be confirmed in studies with larger samples and sufficient numbers of outcome events across predictor categories.

### 4.8. Implications

The findings of this study have significant implications for public health policy and practice in Malaysia and other aging countries. The study identifies population groups at greater risk of digital exclusion by examining sociodemographic characteristics associated with eHealth literacy among older adults. These findings can guide the development of age-appropriate interventions and targeted digital literacy programs to enhance older adults’ ability to access, evaluate, and use digital health information. From a policy perspective, the findings can support the Ministry of Health Malaysia and other stakeholders in improving equitable access to digital health services, mainly as telehealth and mobile health applications become more widely used. The findings also offer guidance for developing more user-friendly digital health platforms tailored to accommodate the needs and limitations of older users.

This study is also relevant in the era of artificial intelligence (AI), as AI-driven health technologies are increasingly being incorporated into self-management and healthcare delivery. AI-enabled tools, such as wearable devices, remote monitoring systems, virtual health assistants, and AI-generated health information platforms, may require users to have appropriate skills to interpret, evaluate, and apply health-related information. Therefore, improving eHealth literacy can improve older adults’ readiness to benefit from AI-driven healthcare innovations and reduce the risk of digital inequalities. The findings are also relevant to the emerging use of artificial intelligence (AI) in healthcare. Although AI literacy was not directly assessed in this study, improving general eHealth literacy may represent one component of preparing older adults for increasingly digital and AI-mediated healthcare environments. Future research should examine AI-specific competencies, including the ability to understand the capabilities and limitations of AI systems, critically evaluate AI-generated health information, and identify potential inaccuracies or biases. Such research would complement the present findings by determining whether the sociodemographic disparities observed in general perceived eHealth literacy also extend to AI-specific health literacy.

Overall, the results may also be applicable to other countries with aging populations, encouraging the development of inclusive digital health initiatives that support healthy aging in an increasingly digital and AI-driven healthcare environment.

## 5. Conclusions

This study examines the level of eHealth literacy and its associated factors among older adults residing in Klang Valley, Malaysia. In summary, eHealth literacy levels were significantly associated with several sociodemographic characteristics, mainly age, education level, and average household income, highlighting the persistence of digital and social inequalities within this population. These findings emphasize the need for targeted, age-appropriate interventions that focus on improving digital skills and confidence among older adults.

In the context of rapidly expanding digital health services and artificial intelligence (AI)-enabled healthcare systems, these findings highlight the importance of understanding inequalities in general eHealth literacy among older adults. Improving eHealth literacy can empower older adults to interact effectively with AI-supported health platforms, support informed health decision-making, and reduce disparities in access to digital healthcare innovations. Targeted, age-appropriate public health interventions, inclusive digital design, and supportive policies are essential to ensure that technological advancements contribute to reducing health inequalities.

Moreover, because the eHEALS assesses perceived general eHealth literacy rather than AI-specific competencies, the present findings do not establish older adults’ readiness to evaluate or use AI-generated health information. Future research should therefore investigate AI-specific health literacy and objectively assessed digital competencies to determine whether existing sociodemographic inequalities in eHealth literacy extend to emerging AI-enabled healthcare environments.

## Figures and Tables

**Table 1:** Sociodemographic characteristics of the study participants (n = 200).

Sociodemographic Characteristic	Category	N (%)
Age		
	60–69 years old	144 (72%)
	70–79 years old	49 (24.5%)
	≥80 years old	7 (3.5%)
Gender		
	Male	80 (40%)
	Female	120 (60%)
Ethnicity		
	Malay	105 (52.5%)
	Chinese	62 (31%)
	Indian	30 (15%)
	Aboriginal	-
	Other	3 (1.5%)
High academic qualifications		
	No educational background	7 (3.5%)
	Primary school	7 (3.5%)
	Secondary school	55 (27.5%)
	Diploma	43 (21.5%)
	Degree	57 (28.5%)
	Master’s	25 (12.5%)
	PHD	6 (3%)
Occupation status		
	Full-time employment	31 (15.5%)
	Part-time employment	7 (3.5%)
	Self-employed	16 (8%)
	Housewife	23 (11.5%)
	Unemployed	10 (5%)
	Retired	112 (56%)
	Other	1 (0.5%)
Main job before retiring (applicable to retired participants only, n = 112)		
	Professional/managerial	66 (33%)
	Technical/skilled worker	6 (3%)
	Clerical/administrative	26 (13%)
	Sales/service worker	1 (0.5%)
	Agricultural/fishery worker	-
	Production/machine operator	2 (1.0%)
	Unskilled labor	6 (3%)
	Self-employed	1 (0.5%)
	Other	4 (2%)
Average monthly household income		
	<RM2500	40 (20%)
	RM2500–RM4849	60 (30%)
	RM4850–RM10,959	62 (31%)
	≥RM10,960	38 (19%)
Receiving a pension		
	Yes	73 (36.5%)
	No	127 (63.5%)
Living arrangements		
	Living alone	31 (15.5%)
	Living with a spouse only	65 (32.5%)
	Living with children or other family or relatives	101 (50.5%)
	Living with caregivers	3 (1.5%)
Health conditions (multiple responses allowed, n = 303)		
	Diabetes	53 (26.5%)
	High blood pressure (hypertension)	86 (43%)
	Heart disease	21 (10.5%)
	High cholesterol	73 (36.5%)
	Cancer	5 (2.5%)
	Chronic respiratory disease (e.g., asthma, COPD)	3 (1.5%)
	None	62 (31%)
	Other	-
Spoken language (multiple responses allowed, n = 475)		
	Malay	188 (94%)
	English	165 (82.5%)
	Mandrin	49 (24.5%)
	Tamil	28 (14%)
	Other Chinese dialects (e.g., Cantonese, Hokkien)	40 (20%)
	Other	5 (2.5%)
English proficiency		
	Not at all proficient	16 (8%)
	Basic	31 (15.5%)
	Intermediate	77 (38.5%)
	Advanced	76 (38%)

Note: Health conditions and spoken language were assessed using multiple-response questions. Participants could select more than one option; therefore, percentages are calculated based on the total sample size (n = 200) and do not sum to 100%. Additionally, only retired participants responded to the question on main job before retiring (n = 112). Non-retired participants were not required to answer this question.

**Table 2:** Descriptive statistics of eHealth Literacy Scale (eHEALS) scores.

	Mean	±Standard Deviation	Min	Max
e-HEALS Score (8–40)	27.29	7.61	8	40

**Table 3:** Distribution of eHealth literacy levels among participants.

e-Health Literacy Levels	Frequency (N = 200)	Percentage (%)
Low (8–20)	33	16.5%
Moderate (21–30)	82	41%
High (31–40)	85	42.5%
Total	200	100%

**Table 4:** Association between sociodemographic factors and eHealth literacy among older people.

Sociodemographic Characteristic	Low (N1 = 33) n1 (%)	Moderate (N2 = 82) n2 (%)	High (N = 85) n3 (%)	*p*-Value
Age				
60–69 years old	16 (11.1%)	63 (43.8%)	65 (45.1%)	0.002 *
70–79 years old	13 (26.5%)	16 (32.7%)	20 (40.8%)	
≥80	4 (57.1%)	3 (42.9%)	0 (0%)	
Gender				
Male	14 (17.5%)	28 (35%)	38 (47.5%)	0.36
Female	19 (15.8%)	54 (45%)	47 (39.2%)	
Ethnicity				
Malay	12 (11.4%)	49 (46.7%)	44 (41.9%)	0.04 *
Chinese	9 (14.5%)	23 (37.1%)	30 (48.4%)	
Indian	11 (36.7%)	9 (30%)	10 (33.3%)	
Other	1 (33.3%)	1 (33.3%)	1 (33.3%)	
Highest academic qualifications				
No educational background	4 (57.1%)	2 (28.6%)	1 (14.3%)	<0.001 *
Primary school	3 (42.9%)	2 (28.6%)	2 (28.6%)	
Secondary school	12 (21.8%)	31 (56.4%)	12 (21.8%)	
Diploma	7 (16.3%)	18 (41.9%)	18 (41.9%)	
Degree	5 (8.8%)	24 (42.1%)	28 (49.1%)	
Postgraduate (master’s/PhD)	2 (6.5%)	5 (16.1%)	24 (77.4%)	
Occupation status				
Full-time employment	5 (16.1%)	11 (35.5%)	15 (48.4%)	0.85
Part-time employment	1 (14.3%)	3 (42.9%)	3 (42.9%)	
Self-employed	3 (18.8%)	7 (43.8%)	6 (37.5%)	
Housewife	5 (21.7%)	13 (56.5%)	5 (21.7%)	
Unemployed	1 (10%)	5 (50%)	4 (40%)	
Retired	18 (16.1%)	43 (38.4%)	51 (45.5%)	
Other	0 (0%)	0 (0%)	1 (100%)	
Main job before retiring				
Professional/technical/clerical	11 (11.2%)	40 (40.8%)	47 (48%)	<0.001 *
Sales/agriculture/production	2 (66.7%)	1 (33.3%)	0 (0%)	
Unskilled	5 (83.3%)	1 (16.7%)	0 (0%)	
Self-employed/other	0 (0%)	1 (20%)	4 (80%)	
Average monthly household income				
<RM2500	15 (37.5%)	10 (25%)	15 (37.5%)	<0.001 *
RM2500–RM4849	10 (16.7%)	34 (56.7%)	16 (26.7%)	
RM4850–RM10,959	6 (9.7%)	29 (46.8%)	27 (43.5%)	
≥RM10,960	2 (5.3%)	9 (23.7%)	27 (71.1%)	
Do you currently receive a pension?				
Yes	12 (16.4%)	32 (43.8%)	29 (39.7%)	0.80
No	21 (16.5%)	50 (39.4%)	56 (44.1%)	
Living arrangements				
Living alone	9 (29%)	12 (38.7%)	10 (32.3%)	0.40
Living with a spouse only	8 (12.3%)	30 (46.2%)	27 (41.5%)	
Living with children or other family or relatives	15 (14.9%)	39 (38.6%)	47 (46.5%)	
Living with caregivers	1 (33.3%)	1 (33.3%)	1 (33.3%)	
Do you currently have any of the following chronic health conditions?				
No chronic disease	11 (17.7%)	24 (28.7%)	27 (43.5%)	0.89
At least one disease	22 (15.9%)	58 (42%)	58 (42%)	
Language ability				
No English	16 (45.7%)	10 (28.6%)	9 (25.7%)	<0.001 *
English (alone or with other languages)	17 (10.3%)	72 (43.6%)	76 (46.1%)	
How would you rate your proficiency in English?				
Not at all proficient	11 (68.8%)	3 (18.8%)	2 (12.5%)	<0.001 *
Basic	6 (19.4%)	15 (48.4%)	10 (32.3%)	
Intermediate	11 (14.3%)	37 (48.1%)	29 (37.7%)	
Advanced	5 (6.6%)	27 (35.5%)	44 (57.9%)	

* Here, *p*-value of <0.05 was considered significant.

**Table 5:** Binary and multivariable logistic regression analyses of factors associated with eHealth literacy correlates of the outcome.

Correlates of the Outcome (Categorical)	Category	Binary Logistic Regression			Multivariable Logistic Regression		
		B (Unadjusted)	COR (95% C.I.)	*p* Value	B (Adjusted)	AOR (95% C.I.)	*p* Value
Age group (yrs)	60–69 yrs	-	1.00	-	-	-	-
	≥70	1.24	3.48 (1.61–7.54)	0.001 *	1.79	6.02 (1.14–31.57)	0.03 *
Gender +	Male	-	1.00	-	-	-	-
	Female	−0.12	0.88 (0.41–1.89)	0.75	-	-	-
Ethnicity	Malay	-	1.00	-	-	-	-
	Chinese	0.27	1.31 (0.52–3.38)	0.56	−0.66	0.51 (0.08–3.01)	0.46
	Indian/other	1.48	4.42 (1.74–11.22)	0.002 *	1.68	5.38 (0.90–31.99)	0.06
Highest academic qualifications	≤Secondary education	-	1.00	-	-	-	-
	Tertiary education	−1.15	0.31 (0.14–0.67)	0.003 *	2.23	9.28 (1.12–76.35)	0.03 *
Occupation status +	Not currently working	-	1.00	-	-	-	-
	Currently working	0.01	1.01 (0.43–2.35)	0.96	-	-	-
Main job before retiring	Professional/skilled	-					
	Non-professional/unskilled/other	0.77	2.17 (0.99–4.76)	0.05 *	0.10	1.10 (0.25–4.84)	0.89
Average monthly household income	<RM2500	-	1.00		-	-	-
	RM2500–RM4849	−1.09	0.33 (0.13–0.84)	0.02 *	−0.55	0.57 (0.09–3.48)	0.54
	RM4850–RM10,959	−1.72	0.17 (0.06–0.51)	0.001 *	−0.47	0.62 (0.05–6.57)	0.69
	≥RM10,960	−2.38	0.09 (0.01–0.44)	0.003 *	−3.63	0.02 (0.001–0.53)	0.01 *
Receiving a pension +	Yes	-	1.00	-	-	-	-
	No	0.007	1.007 (0.46–2.18)	0.98	-	-	-
Living arrangements	Living alone	-	1.00	-			
	Living with a spouse only	−1.07	0.34 (0.11–1.00)	0.05 *	0.17	1.18 (0.14–9.45)	0.87
	Living with family/caregivers	−0.81	0.44 (0.17–1.13)	0.09 *	1.44	4.23 (0.48–36.94)	0.19
Language ability	No English	-	1.00		-	-	-
	English (alone or with other languages)	−1.99	0.13 (0.05–0.31)	<0.001 *	−1.90	0.14 (0.01–1.46)	0.10
English proficiency	Not at all proficient	-	1.00	-	-	-	-
	Basic	−2.21	0.10 (0.02–0.43)	0.002 *	−1.98	0.13 (0.005–3.90)	0.24
	Intermediate	−2.58	0.07 (0.02–0.26)	<0.001 *	−0.97	0.37 (0.009–15.11)	0.60
	Advanced	−3.44	0.03 (0.008–0.12)	<0.001 *	−2.03	0.13 (0.002–8.12)	0.33

* Here, *p*-value of <0.05 was considered significant. + Variables with *p* > 0.25 in the binary logistic regression and therefore not included in the multivariable logistic regression model.

## Data Availability

Data are available on request to the corresponding author due to privacy reasons.

## Supplementary Materials

The following supporting information can be downloaded at: https://www.mdpi.com/article/10.3390/healthcare14183129/s1.
