# Supplementary material for: Factors Associated with e-Health Literacy Among Older Adults in Klang Valley, Malaysia: A Cross-Sectional Survey
Source: Healthcare (Basel). 2026 Sep 21;14(18):3129. doi: 10.3390/healthcare14183129 (PMC13606305; doi:10.3390/healthcare14183129)
Supplement: Supplementary file 1 [file healthcare-14-03129-s001.zip › healthcare-4491239-supplementary.pdf]

Supplementary Materials

Table S1. Sensitivity analysis: crude and adjusted linear regression of sociodemographic factors on the continuous eHealth Literacy Scale (eHEALS) total score among older adults in Klang Valley, Malaysia (n = 200).

Variable	Crude (unadjusted) model		Adjusted multivariable model	
	B (95% CI)	p	B (95% CI)	p
Age (ref: 60–69 years); ≥ 70 years	-4.60 (-6.88 to -2.32)	**<0.001**	-2.77 (-4.97 to -0.57)	**0.014**
Gender (ref: male); female	-0.59 (-2.76 to 1.58)	0.591	-0.40 (-2.43 to 1.62)	0.697
Ethnicity (Malay / Chinese / Indian or other, coded 1–3)	-1.95 (-3.35 to -0.56)	**0.006**	-1.62 (-2.88 to -0.36)	**0.012**
Education (ref: ≤ secondary); tertiary (diploma and above)	6.27 (4.21 to 8.33)	**<0.001**	1.28 (-1.14 to 3.70)	0.297
Occupation status (ref: not currently working); currently working	-0.28 (-2.67 to 2.12)	0.820	-0.60 (-3.35 to 2.15)	0.670
Main job before retiring (ref: professional / skilled); non-professional / unskilled	-2.72 (-4.82 to -0.63)	**0.011**	-0.94 (-3.43 to 1.55)	0.457
Average monthly household income (coded 1–4, increasing)	2.76 (1.78 to 3.73)	**<0.001**	1.03 (-0.04 to 2.10)	0.058
Receiving a pension (ref: yes); no	-0.12 (-2.33 to 2.09)	0.916	1.15 (-0.85 to 3.15)	0.257
Living arrangement (alone / with spouse / with family or caregivers, coded 1–3)	1.10 (-0.34 to 2.54)	0.132	0.08 (-1.19 to 1.35)	0.901
Language ability (ref: no English); English alone or with other languages	7.49 (4.90 to 10.09)	**<0.001**	2.75 (-0.28 to 5.79)	0.075
English proficiency level (coded 1–4, increasing)	4.10 (3.10 to 5.10)	**<0.001**	2.25 (0.77 to 3.72)	**0.003**

B = unstandardised regression coefficient, expressed as the change in eHEALS total score (range 8–40); CI = confidence interval. Crude estimates are from separate simple linear regression models, one per variable. Adjusted estimates are from a single multivariable linear regression model entering all listed variables simultaneously.

Adjusted model: R = 0.599, R^2 = 0.359, adjusted R^2 = 0.321, standard error of the estimate = 6.27; F(11, 188) = 9.55, p <0.001; intercept = 15.84 (95% CI 7.99 to 23.69). No multicollinearity was detected (all tolerance ≥ 0.41; all variance inflation factors ≤ 2.42).

Positive coefficients indicate a higher eHEALS score, i.e. higher perceived eHealth literacy. Ethnicity, household income, living arrangement and English proficiency were entered as single ordered variables with the category order shown.

Bold p-values indicate statistical significance at p < 0.05.
